# Anticoagulant Related Nephropathy Only Partially Develops in C57BL/6 Mice: Hematuria Is Not Accompanied by Red Blood Cell Casts in the Kidney

**DOI:** 10.3389/fmed.2020.617786

**Published:** 2021-02-01

**Authors:** Ajay K. Medipally, Min Xiao, Shahzeb Qaisar, Anjali A. Satoskar, Iouri Ivanov, Brad Rovin, Sergey V. Brodsky

**Affiliations:** ^1^Department of Pathology, The Ohio State University Wexner Medical Center, Columbus, OH, United States; ^2^Department of Medicine, The Ohio State University Wexner Medical Center, Columbus, OH, United States

**Keywords:** anticoagulant related nephropathy, acute kidney injury, mouse model, 5/6 nephrectomy, anticoagulation

## Abstract

Anticoagulant-related nephropathy (ARN) may develop in patients that are on anticoagulation therapy. Rats with 5/6 nephrectomy treated with different anticoagulants showed acute kidney injury (AKI) and red blood cell (RBC) casts in the tubules similar to ARN in humans. The aim of the current study was to investigate the feasibility of inducing ARN in mice. C57BL/6 5/6 nephrectomy mice were treated with warfarin and dabigatran 3 weeks after ablative surgery for 7 days. Two doses of each anticoagulant were used. All anticoagulants resulted in serum creatinine and hematuria increase. Mortality was 63% in 5.0 mg/kg/day of warfarin but only 13% in 2.5 mg/kg/day of warfarin or in 400 mg/kg/day of dabigatran and 0% in 200 mg/kg/day of dabigatran. In spite of increasing hematuria, RBC tubular casts were not seen in mice treated with any anticoagulant. The 5/6 nephrectomy murine model in C57BL/6 mice only partially reproduced ARN in terms of increasing serum creatinine and hematuria, but there were no RBC tubular casts in the remnant kidney.

## Introduction

After our first description of warfarin-related nephropathy [later defined as anticoagulant-related nephropathy (ARN)] in humans (1, 2), we developed an animal model in rats that has close fidelity to the human disease (3, 4). Rats with 5/6 nephrectomy developed glomerular hemorrhage, red blood cell (RBC) tubular casts, and acute kidney injury (AKI) when treated with vitamin K antagonists or a thrombin inhibitor (3, 5). The mechanisms of this AKI include disruption of the glomerular filtration barrier that allows RBC crossing into the urine, increased oxidative stress in the kidney, and acute tubular necrosis (6). The rat model is useful to evaluate the morphological changes in the kidney associated with anticoagulation, but it is difficult to study molecular mechanisms that lead to the glomerular filtration barrier disruption. A murine model could be more useful to study the pathogenesis of ARN because of easier knockout of different genes in mice as compared to rats. The aim of the current study was to investigate the feasibility of ARN induction in C57BL/6 mice.

## Materials and Methods

These studies were approved by the Institutional Animal Care and Use Committees (IACUC) at the Ohio State University.

C57BL/6 mice were obtained from the Charles River Laboratories (Wilmington, MA). A 5/6 nephrectomy was performed in 25–30 g mice as we described previously for rats (3). Briefly, mice were anesthetized with isoflurane/oxygen (1:5), a middle laparotomy was performed, the right kidney was removed, as well as 2/3 of the left kidney at the same time. Kidney tissue from the nephrectomy was frozen at −80°C for further studies. Hemostasis was achieved by hemostatic sponges (Quick clot; Z-medica Corporation, Wallingford, CT). The incision was closed with 4.0 proline, and the animals were kept on a 12/12 h light/dark cycle and on the standard rodent diet with free access to water.

Three weeks later, treatment with an anticoagulant was begun, and daily blood and urine samples were collected. Warfarin sodium (Sigma-Aldrich, St. Louis, MO) and dabigatran etexilate (Boehringer Ingelheim Pharmaceuticals, Inc., Ridgefield, CT) were given once a day *per os via* gavage. Animals were sacrificed on day 7 of the treatment; the remnant kidney was dissected for histological and molecular studies. The histology of the kidney was evaluated on 2–3 mcm sections of paraffin-embedded tissue stained with hematoxylin-eosinophil.

Serum creatinine was measured based on the Jaffe reaction using a creatinine reagent assay (Pointe Scientific, Inc., Canton, MI) as we described earlier. Briefly, 10 μL of serum was mixed with 200 μL of working reagent at 37°C in a 96-well plate, and the absorbance was read at 510 nm at 40 and 100 s using a Molecular Devices Versa Max plate reader (Molecular Devices, Sunnyvale, CA).

Hematuria and proteinuria were evaluated by dipsticks (Siemens reagent strips; Tarrytown, NY) and expressed in a semiquantitative scale from 0 to 3, where 0 is absent, 1 is mild, 2 is moderate, and 3 is severe.

Coagulation parameters [prothrombin time (PT) and activated partial thromboplastin time (aPTT)] were measured using the Biobase coagulation analyzer (model COA01; Genprice Inc., San Jose, CA) based on the manufacturer's protocol. Briefly, blood was collected into a tube containing 3.8% sodium citrate in a ratio of 9:1. The blood was centrifuged at 3,500 RPM for 10 min. Twenty microliters of plasma was placed in the incubation station with 20 μL of the aPTT reagent (Fisher Scientific, Middletown, VA). Then, after 3 min, preheated at 37°C for 10 min and 20 μL of 0.025 M calcium chloride was added. Clotting time was recorded in seconds. For PT, 20 μL of plasma was placed in the incubation station; after 2 min, preheated at 37°C for 10 min and 40 μL of PT reagent (Fisher scientific, Middletown, VA) was added. Clotting time was recorded in seconds. sINR was calculated as changes of PT to the “standard” PT (mean PT calculated from all baselines measurements from all groups), as we described earlier (3).

### Statistical Analysis

Descriptive statistics were used to analyze differences between experimental groups. Data are presented as mean ± standard deviation (SD), unless otherwise specified. Student's two-tailed *t-*test was used to analyze differences between two different time points within the same animal group; one-way ANOVA was used to analyze dynamic changes associated with the treatment. Survival plots were built by using Kaplan–Meier curves. Survival curves were compared by using the Mantel–Haenszel (logrank) test.

## Results

### Changes in Coagulation and Mortality

**Figure 1:**
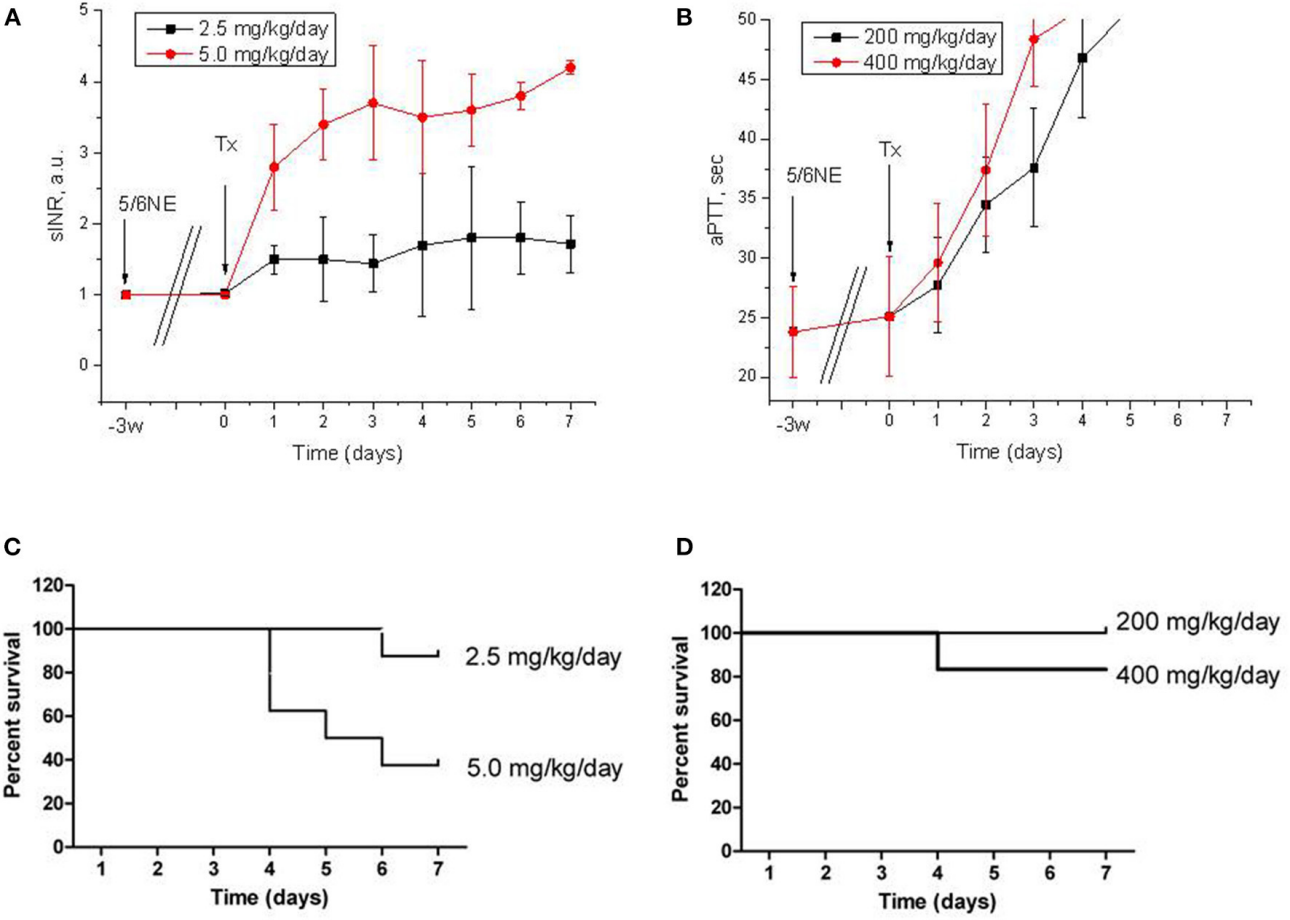
Anticoagulation effects and mortality of warfarin and dabigatran treatments in 5/6 nephrectomy mice. **(A,B)** Changes in prothrombin time calculated as sINR in 5/6 nephrectomy mice treated with 2.5 mg/kg/day (*n* = 8) and 5.0 mg/kg/day (*n* = 8) of warfarin **(A)** or with 200 mg/kg/day (*n* = 7) and 400 mg/kg/day (*n* = 6) of dabigatran **(B)**. **(C,D)** Kaplan-Meier curves of mortality rate in 5/6 nephrectomy mice treated with 2.5 mg/kg/day (*n* = 8) and 5.0 mg/kg/day (*n* = 8) of warfarin **(C)** or with 200 mg/kg/day (*n* = 7) and 400 mg/kg/day (*n* = 6) of dabigatran **(D)**. 5/6NE, 5/6 nephrectomy; Tx, beginning of treatment.

C57BL/6 mice were subjected to ablative surgery (5/6 nephrectomy) and treated with warfarin and dabigatran 3 weeks after the surgery as described in Materials and Methods. Both warfarin and dabigatran in mice had anticoagulation effects that are similar to humans. Thus, treatment with 5.0 mg/kg/day of warfarin resulted in a rapid increase in sINR above 3 a.u. by day 3 of treatment and remained elevated above 3 a.u. until the end of the study (Figure 1A). Treatment with 2.5 mg/kg/day of warfarin resulted in a modest elevation in sINR, and it was in the range 1.5–2 a.u. since day 4 (Figure 1A). Dabigatran increased aPTT above 50 s (upper limit of the reading range for the coagulometer) on day 3 for 400 mg/kg/day and day 4 for 200 mg/kg/day (Figure 1B). Mortality in warfarin-treated animals was high in the 5.0 mg/kg/day group (62.5% of mice died by day 7), whereas it was modest in the 2.5 mg/kg/day group (12.5% died by day 7, *p* = 0.0311) (Figure 1C). Among animals treated with dabigatran, only one mouse died at day 4 in the 400 mg/kg/day group, whereas all mice survived in the 200 mg/kg/day group after 7 days (*p* = 0.2801) (Figure 1D). The main cause of death was hemorrhage to the gastrointestinal tract; no gross evidence of intracranial hemorrhage was noted.

### Changes in Serum Creatinine and Hematuria

**Figure 2:**
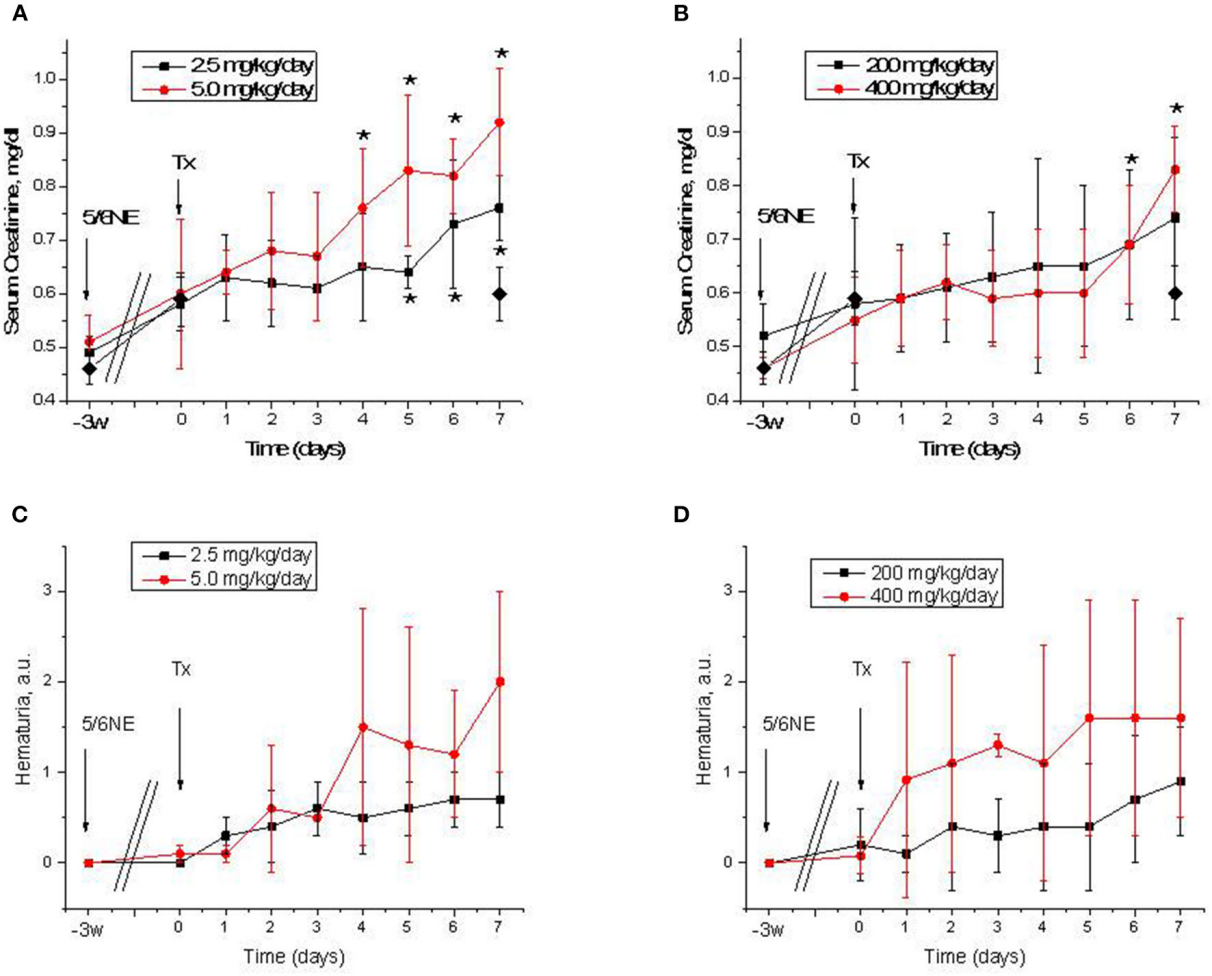
Serum creatinine changes and hematuria in 5/6 nephrectomy mice treated with warfarin and dabigatran. **(A,B)** Serum creatinine changes in 5/6 nephrectomy mice treated with 2.5 mg/kg/day (*n* = 8) and 5.0 mg/kg/day (*n* = 8) of warfarin **(A)** or with 200 mg/kg/day (*n* = 7) and 400 mg/kg/day (*n* = 6) of dabigatran **(B)**. Control (vehicle-treated group) data on day 0 and 7 are shown in a solid diamond on both **(A,B)**. **(C,D)** Hematuria in 5/6 nephrectomy mice treated with 2.5 mg/kg/day (*n* = 8) and 5.0 mg/kg/day (*n* = 8) of warfarin **(C)** or with 200 mg/kg/day (*n* = 7) and 400 mg/kg/day (*n* = 6) of dabigatran **(D)**. 5/6NE, 5/6 nephrectomy; Tx, beginning of treatment. *p < 0.05 as compared to day 0 of treatment. Hematuria was quantitated by a semiquantitative scale from 0 to 3, where 0 is absent, 1 is mild, 2 is moderate, and 3 is severe.

All mice had an increase in serum creatinine after 5/6 nephrectomy (from 0.50 ± 0.04 to 0.58 ± 0.1 mg/dL 3 weeks after the surgery, *p* = 0.004). In mice treated with warfarin, serum creatinine was increased in both groups. Treatment with 2.5 mg/kg/day resulted in a significant increase in serum creatinine by day 5 of treatment (0.64 ± 0.03 mg/dL, *p* = 0.009). Treatment with 5.0 mg/kg/day resulted in a more rapid increase in serum creatinine (significantly elevated at day 4 of treatment, 0.76 ± 0.11 mg/dL, *p* = 0.042) (Figure 2A). In control (vehicle-treated) mice, serum creatinine remained unchanged 7 days after the treatment (0.60 ± 0.05 mg/dL). Simultaneously with the increase in serum creatinine, there was an increase in hematuria that was more prominent in mice treated with 5 mg/kg/day of warfarin (Figure 2C). In the control (vehicle-treated) group, hematuria did not change by day 7 (0.1 ± 0.11 and 0.1 ± 0.21 a.u. days 0 and 7, respectively).

Similarly to warfarin, treatment with dabigatran resulted in an increase in serum creatinine in 5/6 nephrectomy mice (Figure 2B). Both 200 and 400 mg/kg/day of dabigatran resulted in serum creatinine increase, but only with 400 mg/kg/day of dabigatran was such increase significant at day 6 (0.69 ± 0.11 mg/dL, *p* = 0.0355), whereas serum creatinine elevation in mice treated with 200 mg/kg/day of dabigatran was not significant (Figure 2B). Hematuria was increased in both dabigatran treatment groups (Figure 2D).

### Morphologic Changes in the Kidney

Morphological changes in the kidney in mice treated with both anticoagulants included mild acute tubular epithelial cell injury (more pronounced in high dosage groups), but no RBC casts in the tubules or RBC in the Bowman's space were seen.

## Discussion

Since our kidney biopsy findings in patients treated with warfarin were described over 10 years ago (2), many cases of ARN in humans have since been reported (7). The pathogenesis of this condition is unclear, and it requires further studies. We had demonstrated that 5/6 nephrectomy rats treated with anticoagulants (warfarin and dabigatran) 3 weeks after the ablative surgery have an increase in serum creatinine and morphological changes in the kidney that are similar to the human disease (3, 5). Unfortunately, it is not easy to control gene and protein expression in rats; therefore, there is a need for a murine model of ARN. Here, we report our findings when we treated C57BL/6 5/6 nephrectomy mice with warfarin and dabigatran.

Our data indicate that C57BL/6 mice require higher doses of anticoagulants as compared to rats, which corresponds to literature data (8). Thus, in our previous works, we used 0.75 mg/kg/day of warfarin and 50 mg/kg/day of dabigatran in rats, and we achieved anticoagulation levels similar to those in humans. These treatments resulted in ARN in rats with an increase in serum creatinine and RBC casts in the tubules. In mice, we had to use 2.5 mg/kg/day of warfarin to increase PT two-fold and 5.0 mg/kg/day to increase PT four times and higher. The high warfarin dose was fatal for mice, and there was over 50% mortality (Figure 1C). Similarly, dabigatran in the dose of 200 mg/kg/day increased aPTT two-fold in mice (Figure 1B), whereas in rats a similar effect was achieved with only 50 mg/kg/day (5). Effects on the kidney function were different in mice and rats. Even though there was the increase in serum creatinine and hematuria which were associated with treatment with both anticoagulants in C57BL/6 mice, the morphological hallmark of ARN such as occlusive red blood casts in the tubules was lacking in mice but was present in rats. One possible explanation of such a difference could be related to the fact that mice are more resistant to 5/6 nephrectomy compared to rats and the decline in kidney function in C57BL/6 mice is less pronounced than in other mouse strains (9). Even though we observed a significant increase in serum creatinine 3 weeks after the ablative surgery in C57BL/6 mice, the kidney function was probably still not impaired enough to develop RBC casts in the tubules. Other mouse strains, such as BALB/c mice, could be more susceptible to developing ARN, but since many knockout mice are developed based on the C57BL/6 strain, it is desirable to develop a model to study ARN using the C57BL/6 mice. One possible solution would be to induce hypertension by using angiotensin II in C57BL/6 mice after 5/6 nephrectomy and to study ARN after the treatment; this requires further investigation (9). However, even in the absence of occlusive tubular RBC casts, we achieved a dose-dependent increase in serum creatinine and hematuria, indicating that C57BL/6 5/6 nephrectomy mice, at least partially, could be used to study ARN.

## Data Availability Statement

The raw data supporting the conclusions of this article will be made available by the authors, without undue reservation.

## Ethics Statement

The animal study was reviewed and approved by the Institutional Animal Care and Use Committees (IACUC) at the Ohio State University.

## Author Contributions

AM conducted animals studies (surgeries), collected and analyzed samples, and participated in data analysis, writing, and reviewing the manuscript. MX conducted animals studies, collected and analyzed samples, and participated in data analysis, writing, and reviewing the manuscript. SQ collected and analyzed samples, participated in data analysis, writing, and reviewing the manuscript. AS, II, and BR participated in study design, data analysis, writing, and reviewing the manuscript. SB oversees the entire study, designed experiments, and performed data analysis, writing, and reviewing the manuscript. All authors contributed to the article and approved the submitted version.

## Conflict of Interest

SB received a grant from NIH (NIDDK grant R01DK117102). The remaining authors declare that the research was conducted in the absence of any commercial or financial relationships that could be construed as a potential conflict of interest.
